# Supplementary material for: Pyruvate Dehydrogenase Kinase 1 inhibition mediated oxidative phosphorylation enhancement in cartilage promotes osteoarthritis progression
Source: BMC Musculoskelet Disord. 2023 Jul 20;24:597. doi: 10.1186/s12891-023-06585-6 (PMC10357736; doi:10.1186/s12891-023-06585-6)
Supplement: Supplementary file 1 — Additional file 1. [file 12891_2023_6585_MOESM1_ESM.zip › KEGG.Gsea.1653622432464/KEGG_FOCAL_ADHESION.html]

Details for gene set KEGG\_FOCAL\_ADHESION[GSEA]

|  || Dataset | 55457\_collapsed\_to\_symbols.cls.cls #OA\_versus\_normal.cls.cls #OA\_versus\_normal\_repos |
| Phenotype | cls.cls#OA\_versus\_normal\_repos |
| Upregulated in class | normal |
| GeneSet | KEGG\_FOCAL\_ADHESION |
| Enrichment Score (ES) | -0.31755656 |
| Normalized Enrichment Score (NES) | -1.5098504 |
| Nominal p-value | 0.0015037594 |
| FDR q-value | 0.23544656 |
| FWER p-Value | 0.885 |
Table: GSEA Results Summary

  

Fig 1: Enrichment plot: KEGG\_FOCAL\_ADHESION      
 Profile of the Running ES Score & Positions of GeneSet Members on the Rank Ordered List

  

| SYMBOL | TITLE | RANK IN GENE LIST | RANK METRIC SCORE | RUNNING ES | CORE ENRICHMENT || 1 | PDGFC | platelet derived growth factor C [Source:HGNC Symbol;Acc:HGNC:8801] | 103 | 0.911 | 0.0066 | No |
| 2 | VTN | vitronectin [Source:HGNC Symbol;Acc:HGNC:12724] | 297 | 0.753 | 0.0035 | No |
| 3 | COMP | cartilage oligomeric matrix protein [Source:HGNC Symbol;Acc:HGNC:2227] | 415 | 0.700 | 0.0056 | No |
| 4 | PARVB | parvin beta [Source:HGNC Symbol;Acc:HGNC:14653] | 551 | 0.641 | 0.0053 | No |
| 5 | VAV1 | vav guanine nucleotide exchange factor 1 [Source:HGNC Symbol;Acc:HGNC:12657] | 601 | 0.622 | 0.0114 | No |
| 6 | PPP1CA | protein phosphatase 1 catalytic subunit alpha [Source:HGNC Symbol;Acc:HGNC:9281] | 603 | 0.622 | 0.0213 | No |
| 7 | CHAD | chondroadherin [Source:HGNC Symbol;Acc:HGNC:1909] | 629 | 0.612 | 0.0292 | No |
| 8 | MAPK9 | mitogen-activated protein kinase 9 [Source:HGNC Symbol;Acc:HGNC:6886] | 721 | 0.588 | 0.0315 | No |
| 9 | PPP1CC | protein phosphatase 1 catalytic subunit gamma [Source:HGNC Symbol;Acc:HGNC:9283] | 744 | 0.583 | 0.0392 | No |
| 10 | MAPK3 | mitogen-activated protein kinase 3 [Source:HGNC Symbol;Acc:HGNC:6877] | 757 | 0.579 | 0.0476 | No |
| 11 | ERBB2 | erb-b2 receptor tyrosine kinase 2 [Source:HGNC Symbol;Acc:HGNC:3430] | 963 | 0.526 | 0.0399 | No |
| 12 | AKT1 | AKT serine/threonine kinase 1 [Source:HGNC Symbol;Acc:HGNC:391] | 1192 | 0.478 | 0.0297 | No |
| 13 | ILK | integrin linked kinase [Source:HGNC Symbol;Acc:HGNC:6040] | 1286 | 0.460 | 0.0297 | No |
| 14 | ITGA4 | integrin subunit alpha 4 [Source:HGNC Symbol;Acc:HGNC:6140] | 1854 | 0.379 | -0.0088 | No |
| 15 | CRK | "CRK proto-oncogene, adaptor protein [Source:HGNC Symbol;Acc:HGNC:2362]" | 2018 | 0.360 | -0.0158 | No |
| 16 | COL11A1 | collagen type XI alpha 1 chain [Source:HGNC Symbol;Acc:HGNC:2186] | 2128 | 0.347 | -0.0188 | No |
| 17 | LAMC3 | laminin subunit gamma 3 [Source:HGNC Symbol;Acc:HGNC:6494] | 2177 | 0.341 | -0.0171 | No |
| 18 | PIK3CB | "phosphatidylinositol-4,5-bisphosphate 3-kinase catalytic subunit beta [Source:HGNC Symbol;Acc:HGNC:8976]" | 2186 | 0.340 | -0.0123 | No |
| 19 | VEGFC | vascular endothelial growth factor C [Source:HGNC Symbol;Acc:HGNC:12682] | 2239 | 0.335 | -0.0110 | No |
| 20 | CRKL | "CRK like proto-oncogene, adaptor protein [Source:HGNC Symbol;Acc:HGNC:2363]" | 2283 | 0.329 | -0.0091 | No |
| 21 | IGF1 | insulin like growth factor 1 [Source:HGNC Symbol;Acc:HGNC:5464] | 2286 | 0.328 | -0.0039 | No |
| 22 | THBS4 | thrombospondin 4 [Source:HGNC Symbol;Acc:HGNC:11788] | 2349 | 0.320 | -0.0036 | No |
| 23 | MYL2 | myosin light chain 2 [Source:HGNC Symbol;Acc:HGNC:7583] | 2376 | 0.317 | -0.0006 | No |
| 24 | EGF | epidermal growth factor [Source:HGNC Symbol;Acc:HGNC:3229] | 2399 | 0.315 | 0.0027 | No |
| 25 | ITGA1 | integrin subunit alpha 1 [Source:HGNC Symbol;Acc:HGNC:6134] | 2421 | 0.311 | 0.0061 | No |
| 26 | THBS3 | thrombospondin 3 [Source:HGNC Symbol;Acc:HGNC:11787] | 2474 | 0.305 | 0.0069 | No |
| 27 | SHC1 | SHC adaptor protein 1 [Source:HGNC Symbol;Acc:HGNC:10840] | 2485 | 0.304 | 0.0110 | No |
| 28 | CCND2 | cyclin D2 [Source:HGNC Symbol;Acc:HGNC:1583] | 2508 | 0.302 | 0.0142 | No |
| 29 | ACTN2 | actinin alpha 2 [Source:HGNC Symbol;Acc:HGNC:164] | 2532 | 0.299 | 0.0172 | No |
| 30 | HGF | hepatocyte growth factor [Source:HGNC Symbol;Acc:HGNC:4893] | 2547 | 0.297 | 0.0209 | No |
| 31 | MYL5 | myosin light chain 5 [Source:HGNC Symbol;Acc:HGNC:7586] | 2705 | 0.279 | 0.0130 | No |
| 32 | MYLPF | "myosin light chain, phosphorylatable, fast skeletal muscle [Source:HGNC Symbol;Acc:HGNC:29824]" | 2816 | 0.268 | 0.0087 | No |
| 33 | ITGB5 | integrin subunit beta 5 [Source:HGNC Symbol;Acc:HGNC:6160] | 2823 | 0.267 | 0.0125 | No |
| 34 | PDGFRA | platelet derived growth factor receptor alpha [Source:HGNC Symbol;Acc:HGNC:8803] | 2943 | 0.256 | 0.0073 | No |
| 35 | MYL12A | myosin light chain 12A [Source:HGNC Symbol;Acc:HGNC:16701] | 3039 | 0.246 | 0.0037 | No |
| 36 | ELK1 | ETS transcription factor ELK1 [Source:HGNC Symbol;Acc:HGNC:3321] | 3357 | 0.216 | -0.0177 | No |
| 37 | PARVA | parvin alpha [Source:HGNC Symbol;Acc:HGNC:14652] | 3499 | 0.202 | -0.0256 | No |
| 38 | COL4A4 | collagen type IV alpha 4 chain [Source:HGNC Symbol;Acc:HGNC:2206] | 3592 | 0.195 | -0.0297 | No |
| 39 | LAMB3 | laminin subunit beta 3 [Source:HGNC Symbol;Acc:HGNC:6490] | 3679 | 0.188 | -0.0334 | No |
| 40 | PRKCA | protein kinase C alpha [Source:HGNC Symbol;Acc:HGNC:9393] | 3728 | 0.183 | -0.0342 | No |
| 41 | BAD | BCL2 associated agonist of cell death [Source:HGNC Symbol;Acc:HGNC:936] | 3788 | 0.178 | -0.0360 | No |
| 42 | RAC3 | Rac family small GTPase 3 [Source:HGNC Symbol;Acc:HGNC:9803] | 3849 | 0.171 | -0.0380 | No |
| 43 | PDGFD | platelet derived growth factor D [Source:HGNC Symbol;Acc:HGNC:30620] | 3850 | 0.171 | -0.0352 | No |
| 44 | PAK1 | p21 (RAC1) activated kinase 1 [Source:HGNC Symbol;Acc:HGNC:8590] | 3861 | 0.169 | -0.0333 | No |
| 45 | VAV3 | vav guanine nucleotide exchange factor 3 [Source:HGNC Symbol;Acc:HGNC:12659] | 4082 | 0.148 | -0.0483 | No |
| 46 | MYL10 | myosin light chain 10 [Source:HGNC Symbol;Acc:HGNC:29825] | 4125 | 0.144 | -0.0492 | No |
| 47 | SPP1 | secreted phosphoprotein 1 [Source:HGNC Symbol;Acc:HGNC:11255] | 4205 | 0.137 | -0.0533 | No |
| 48 | CCND3 | cyclin D3 [Source:HGNC Symbol;Acc:HGNC:1585] | 4426 | 0.119 | -0.0686 | No |
| 49 | TNC | tenascin C [Source:HGNC Symbol;Acc:HGNC:5318] | 4874 | 0.082 | -0.1025 | No |
| 50 | CAV2 | caveolin 2 [Source:HGNC Symbol;Acc:HGNC:1528] | 4924 | 0.078 | -0.1051 | No |
| 51 | MYL9 | myosin light chain 9 [Source:HGNC Symbol;Acc:HGNC:15754] | 4939 | 0.077 | -0.1050 | No |
| 52 | THBS2 | thrombospondin 2 [Source:HGNC Symbol;Acc:HGNC:11786] | 4943 | 0.077 | -0.1040 | No |
| 53 | DOCK1 | dedicator of cytokinesis 1 [Source:HGNC Symbol;Acc:HGNC:2987] | 4998 | 0.073 | -0.1070 | No |
| 54 | BIRC2 | baculoviral IAP repeat containing 2 [Source:HGNC Symbol;Acc:HGNC:590] | 5054 | 0.069 | -0.1102 | No |
| 55 | MYLK | myosin light chain kinase [Source:HGNC Symbol;Acc:HGNC:7590] | 5079 | 0.066 | -0.1111 | No |
| 56 | LAMB4 | laminin subunit beta 4 [Source:HGNC Symbol;Acc:HGNC:6491] | 5111 | 0.063 | -0.1125 | No |
| 57 | VASP | vasodilator stimulated phosphoprotein [Source:HGNC Symbol;Acc:HGNC:12652] | 5143 | 0.060 | -0.1140 | No |
| 58 | TLN2 | talin 2 [Source:HGNC Symbol;Acc:HGNC:15447] | 5161 | 0.059 | -0.1144 | No |
| 59 | COL5A2 | collagen type V alpha 2 chain [Source:HGNC Symbol;Acc:HGNC:2210] | 5262 | 0.051 | -0.1214 | No |
| 60 | MYLK3 | myosin light chain kinase 3 [Source:HGNC Symbol;Acc:HGNC:29826] | 5294 | 0.048 | -0.1231 | No |
| 61 | COL5A1 | collagen type V alpha 1 chain [Source:HGNC Symbol;Acc:HGNC:2209] | 5414 | 0.039 | -0.1318 | No |
| 62 | LAMA4 | laminin subunit alpha 4 [Source:HGNC Symbol;Acc:HGNC:6484] | 5473 | 0.035 | -0.1358 | No |
| 63 | BCL2 | BCL2 apoptosis regulator [Source:HGNC Symbol;Acc:HGNC:990] | 5609 | 0.023 | -0.1461 | No |
| 64 | RELN | reelin [Source:HGNC Symbol;Acc:HGNC:9957] | 5635 | 0.021 | -0.1477 | No |
| 65 | GRB2 | growth factor receptor bound protein 2 [Source:HGNC Symbol;Acc:HGNC:4566] | 5697 | 0.016 | -0.1522 | No |
| 66 | SHC2 | SHC adaptor protein 2 [Source:HGNC Symbol;Acc:HGNC:29869] | 5738 | 0.012 | -0.1552 | No |
| 67 | ZYX | zyxin [Source:HGNC Symbol;Acc:HGNC:13200] | 5861 | 0.004 | -0.1647 | No |
| 68 | BIRC3 | baculoviral IAP repeat containing 3 [Source:HGNC Symbol;Acc:HGNC:591] | 5942 | -0.001 | -0.1710 | No |
| 69 | COL4A6 | collagen type IV alpha 6 chain [Source:HGNC Symbol;Acc:HGNC:2208] | 5975 | -0.003 | -0.1734 | No |
| 70 | RAC2 | Rac family small GTPase 2 [Source:HGNC Symbol;Acc:HGNC:9802] | 6098 | -0.013 | -0.1828 | No |
| 71 | RAF1 | "Raf-1 proto-oncogene, serine/threonine kinase [Source:HGNC Symbol;Acc:HGNC:9829]" | 6187 | -0.019 | -0.1895 | No |
| 72 | FLNC | filamin C [Source:HGNC Symbol;Acc:HGNC:3756] | 6408 | -0.036 | -0.2062 | No |
| 73 | PRKCB | protein kinase C beta [Source:HGNC Symbol;Acc:HGNC:9395] | 6453 | -0.039 | -0.2090 | No |
| 74 | FLNB | filamin B [Source:HGNC Symbol;Acc:HGNC:3755] | 6499 | -0.042 | -0.2119 | No |
| 75 | RAP1A | "RAP1A, member of RAS oncogene family [Source:HGNC Symbol;Acc:HGNC:9855]" | 6580 | -0.048 | -0.2174 | No |
| 76 | PIP5K1C | phosphatidylinositol-4-phosphate 5-kinase type 1 gamma [Source:HGNC Symbol;Acc:HGNC:8996] | 6601 | -0.050 | -0.2182 | No |
| 77 | MAPK1 | mitogen-activated protein kinase 1 [Source:HGNC Symbol;Acc:HGNC:6871] | 6853 | -0.068 | -0.2368 | No |
| 78 | PDGFRB | platelet derived growth factor receptor beta [Source:HGNC Symbol;Acc:HGNC:8804] | 6914 | -0.073 | -0.2404 | No |
| 79 | SRC | "SRC proto-oncogene, non-receptor tyrosine kinase [Source:HGNC Symbol;Acc:HGNC:11283]" | 6940 | -0.075 | -0.2411 | No |
| 80 | RAP1B | "RAP1B, member of RAS oncogene family [Source:HGNC Symbol;Acc:HGNC:9857]" | 6980 | -0.078 | -0.2430 | No |
| 81 | ITGAV | integrin subunit alpha V [Source:HGNC Symbol;Acc:HGNC:6150] | 7067 | -0.085 | -0.2483 | No |
| 82 | DIAPH1 | diaphanous related formin 1 [Source:HGNC Symbol;Acc:HGNC:2876] | 7072 | -0.086 | -0.2473 | No |
| 83 | MYL7 | myosin light chain 7 [Source:HGNC Symbol;Acc:HGNC:21719] | 7137 | -0.091 | -0.2508 | No |
| 84 | TNXB | tenascin XB [Source:HGNC Symbol;Acc:HGNC:11976] | 7189 | -0.095 | -0.2533 | No |
| 85 | RAC1 | Rac family small GTPase 1 [Source:HGNC Symbol;Acc:HGNC:9801] | 7192 | -0.096 | -0.2519 | No |
| 86 | CAV1 | caveolin 1 [Source:HGNC Symbol;Acc:HGNC:1527] | 7205 | -0.097 | -0.2513 | No |
| 87 | PTK2 | protein tyrosine kinase 2 [Source:HGNC Symbol;Acc:HGNC:9611] | 7315 | -0.103 | -0.2582 | No |
| 88 | MET | "MET proto-oncogene, receptor tyrosine kinase [Source:HGNC Symbol;Acc:HGNC:7029]" | 7322 | -0.104 | -0.2570 | No |
| 89 | ARHGAP5 | Rho GTPase activating protein 5 [Source:HGNC Symbol;Acc:HGNC:675] | 7357 | -0.106 | -0.2580 | No |
| 90 | LAMA2 | laminin subunit alpha 2 [Source:HGNC Symbol;Acc:HGNC:6482] | 7392 | -0.110 | -0.2589 | No |
| 91 | PIK3CD | "phosphatidylinositol-4,5-bisphosphate 3-kinase catalytic subunit delta [Source:HGNC Symbol;Acc:HGNC:8977]" | 7405 | -0.111 | -0.2581 | No |
| 92 | CCND1 | cyclin D1 [Source:HGNC Symbol;Acc:HGNC:1582] | 7512 | -0.119 | -0.2645 | No |
| 93 | ACTN3 | actinin alpha 3 [Source:HGNC Symbol;Acc:HGNC:165] | 7557 | -0.124 | -0.2660 | No |
| 94 | PAK5 | p21 (RAC1) activated kinase 5 [Source:HGNC Symbol;Acc:HGNC:15916] | 7558 | -0.124 | -0.2640 | No |
| 95 | VCL | vinculin [Source:HGNC Symbol;Acc:HGNC:12665] | 7687 | -0.133 | -0.2719 | No |
| 96 | PIK3CA | "phosphatidylinositol-4,5-bisphosphate 3-kinase catalytic subunit alpha [Source:HGNC Symbol;Acc:HGNC:8975]" | 7733 | -0.136 | -0.2732 | No |
| 97 | PAK2 | p21 (RAC1) activated kinase 2 [Source:HGNC Symbol;Acc:HGNC:8591] | 7762 | -0.139 | -0.2732 | No |
| 98 | ITGA9 | integrin subunit alpha 9 [Source:HGNC Symbol;Acc:HGNC:6145] | 7785 | -0.140 | -0.2727 | No |
| 99 | LAMB2 | laminin subunit beta 2 [Source:HGNC Symbol;Acc:HGNC:6487] | 7841 | -0.144 | -0.2747 | No |
| 100 | ITGA2 | integrin subunit alpha 2 [Source:HGNC Symbol;Acc:HGNC:6137] | 7857 | -0.145 | -0.2735 | No |
| 101 | ITGB7 | integrin subunit beta 7 [Source:HGNC Symbol;Acc:HGNC:6162] | 7873 | -0.146 | -0.2724 | No |
| 102 | PIK3CG | "phosphatidylinositol-4,5-bisphosphate 3-kinase catalytic subunit gamma [Source:HGNC Symbol;Acc:HGNC:8978]" | 7953 | -0.152 | -0.2762 | No |
| 103 | HRAS | "HRas proto-oncogene, GTPase [Source:HGNC Symbol;Acc:HGNC:5173]" | 7977 | -0.153 | -0.2755 | No |
| 104 | AKT3 | AKT serine/threonine kinase 3 [Source:HGNC Symbol;Acc:HGNC:393] | 8008 | -0.156 | -0.2753 | No |
| 105 | SOS2 | SOS Ras/Rho guanine nucleotide exchange factor 2 [Source:HGNC Symbol;Acc:HGNC:11188] | 8058 | -0.160 | -0.2766 | No |
| 106 | CAPN2 | calpain 2 [Source:HGNC Symbol;Acc:HGNC:1479] | 8114 | -0.164 | -0.2783 | No |
| 107 | COL1A1 | collagen type I alpha 1 chain [Source:HGNC Symbol;Acc:HGNC:2197] | 8133 | -0.165 | -0.2771 | No |
| 108 | BRAF | "B-Raf proto-oncogene, serine/threonine kinase [Source:HGNC Symbol;Acc:HGNC:1097]" | 8171 | -0.169 | -0.2773 | No |
| 109 | PAK3 | p21 (RAC1) activated kinase 3 [Source:HGNC Symbol;Acc:HGNC:8592] | 8266 | -0.176 | -0.2818 | No |
| 110 | ACTN1 | actinin alpha 1 [Source:HGNC Symbol;Acc:HGNC:163] | 8385 | -0.184 | -0.2881 | No |
| 111 | PRKCG | protein kinase C gamma [Source:HGNC Symbol;Acc:HGNC:9402] | 8582 | -0.200 | -0.3004 | No |
| 112 | RHOA | ras homolog family member A [Source:HGNC Symbol;Acc:HGNC:667] | 8601 | -0.201 | -0.2985 | No |
| 113 | VEGFB | vascular endothelial growth factor B [Source:HGNC Symbol;Acc:HGNC:12681] | 8673 | -0.206 | -0.3008 | No |
| 114 | TNR | tenascin R [Source:HGNC Symbol;Acc:HGNC:11953] | 8715 | -0.210 | -0.3006 | No |
| 115 | XIAP | X-linked inhibitor of apoptosis [Source:HGNC Symbol;Acc:HGNC:592] | 8750 | -0.212 | -0.2999 | No |
| 116 | COL3A1 | collagen type III alpha 1 chain [Source:HGNC Symbol;Acc:HGNC:2201] | 8784 | -0.215 | -0.2990 | No |
| 117 | PIK3R3 | phosphoinositide-3-kinase regulatory subunit 3 [Source:HGNC Symbol;Acc:HGNC:8981] | 8801 | -0.217 | -0.2968 | No |
| 118 | PIK3R5 | phosphoinositide-3-kinase regulatory subunit 5 [Source:HGNC Symbol;Acc:HGNC:30035] | 8804 | -0.217 | -0.2935 | No |
| 119 | ARHGAP35 | Rho GTPase activating protein 35 [Source:HGNC Symbol;Acc:HGNC:4591] | 8835 | -0.220 | -0.2923 | No |
| 120 | CAV3 | caveolin 3 [Source:HGNC Symbol;Acc:HGNC:1529] | 8953 | -0.228 | -0.2978 | No |
| 121 | MAPK8 | mitogen-activated protein kinase 8 [Source:HGNC Symbol;Acc:HGNC:6881] | 8956 | -0.228 | -0.2943 | No |
| 122 | RASGRF1 | Ras protein specific guanine nucleotide releasing factor 1 [Source:HGNC Symbol;Acc:HGNC:9875] | 8982 | -0.230 | -0.2926 | No |
| 123 | PAK6 | p21 (RAC1) activated kinase 6 [Source:HGNC Symbol;Acc:HGNC:16061] | 9068 | -0.237 | -0.2954 | No |
| 124 | ITGA6 | integrin subunit alpha 6 [Source:HGNC Symbol;Acc:HGNC:6142] | 9112 | -0.240 | -0.2950 | No |
| 125 | TLN1 | talin 1 [Source:HGNC Symbol;Acc:HGNC:11845] | 9131 | -0.242 | -0.2925 | No |
| 126 | VWF | von Willebrand factor [Source:HGNC Symbol;Acc:HGNC:12726] | 9180 | -0.246 | -0.2923 | No |
| 127 | ITGA10 | integrin subunit alpha 10 [Source:HGNC Symbol;Acc:HGNC:6135] | 9296 | -0.256 | -0.2972 | No |
| 128 | ITGB8 | integrin subunit beta 8 [Source:HGNC Symbol;Acc:HGNC:6163] | 9442 | -0.268 | -0.3043 | No |
| 129 | SHC3 | SHC adaptor protein 3 [Source:HGNC Symbol;Acc:HGNC:18181] | 9608 | -0.284 | -0.3127 | Yes |
| 130 | ITGB6 | integrin subunit beta 6 [Source:HGNC Symbol;Acc:HGNC:6161] | 9614 | -0.284 | -0.3086 | Yes |
| 131 | MAPK10 | mitogen-activated protein kinase 10 [Source:HGNC Symbol;Acc:HGNC:6872] | 9699 | -0.291 | -0.3105 | Yes |
| 132 | ITGA7 | integrin subunit alpha 7 [Source:HGNC Symbol;Acc:HGNC:6143] | 9724 | -0.294 | -0.3076 | Yes |
| 133 | COL11A2 | collagen type XI alpha 2 chain [Source:HGNC Symbol;Acc:HGNC:2187] | 9734 | -0.295 | -0.3036 | Yes |
| 134 | PXN | paxillin [Source:HGNC Symbol;Acc:HGNC:9718] | 9845 | -0.305 | -0.3073 | Yes |
| 135 | FLNA | filamin A [Source:HGNC Symbol;Acc:HGNC:3754] | 9976 | -0.318 | -0.3124 | Yes |
| 136 | COL1A2 | collagen type I alpha 2 chain [Source:HGNC Symbol;Acc:HGNC:2198] | 9985 | -0.320 | -0.3079 | Yes |
| 137 | PAK4 | p21 (RAC1) activated kinase 4 [Source:HGNC Symbol;Acc:HGNC:16059] | 10004 | -0.322 | -0.3042 | Yes |
| 138 | FYN | "FYN proto-oncogene, Src family tyrosine kinase [Source:HGNC Symbol;Acc:HGNC:4037]" | 10092 | -0.330 | -0.3057 | Yes |
| 139 | COL2A1 | collagen type II alpha 1 chain [Source:HGNC Symbol;Acc:HGNC:2200] | 10122 | -0.333 | -0.3026 | Yes |
| 140 | ITGB4 | integrin subunit beta 4 [Source:HGNC Symbol;Acc:HGNC:6158] | 10159 | -0.337 | -0.3000 | Yes |
| 141 | MAP2K1 | mitogen-activated protein kinase kinase 1 [Source:HGNC Symbol;Acc:HGNC:6840] | 10197 | -0.340 | -0.2974 | Yes |
| 142 | ITGB3 | integrin subunit beta 3 [Source:HGNC Symbol;Acc:HGNC:6156] | 10310 | -0.352 | -0.3006 | Yes |
| 143 | IBSP | integrin binding sialoprotein [Source:HGNC Symbol;Acc:HGNC:5341] | 10351 | -0.356 | -0.2980 | Yes |
| 144 | COL6A2 | collagen type VI alpha 2 chain [Source:HGNC Symbol;Acc:HGNC:2212] | 10385 | -0.359 | -0.2948 | Yes |
| 145 | COL5A3 | collagen type V alpha 3 chain [Source:HGNC Symbol;Acc:HGNC:14864] | 10456 | -0.368 | -0.2944 | Yes |
| 146 | ACTN4 | actinin alpha 4 [Source:HGNC Symbol;Acc:HGNC:166] | 10472 | -0.370 | -0.2896 | Yes |
| 147 | MYL12B | myosin light chain 12B [Source:HGNC Symbol;Acc:HGNC:29827] | 10505 | -0.374 | -0.2861 | Yes |
| 148 | LAMC2 | laminin subunit gamma 2 [Source:HGNC Symbol;Acc:HGNC:6493] | 10531 | -0.376 | -0.2820 | Yes |
| 149 | LAMC1 | laminin subunit gamma 1 [Source:HGNC Symbol;Acc:HGNC:6492] | 10642 | -0.387 | -0.2845 | Yes |
| 150 | ROCK2 | Rho associated coiled-coil containing protein kinase 2 [Source:HGNC Symbol;Acc:HGNC:10252] | 10648 | -0.388 | -0.2786 | Yes |
| 151 | TNN | tenascin N [Source:HGNC Symbol;Acc:HGNC:22942] | 10713 | -0.395 | -0.2773 | Yes |
| 152 | FLT1 | fms related receptor tyrosine kinase 1 [Source:HGNC Symbol;Acc:HGNC:3763] | 10826 | -0.408 | -0.2795 | Yes |
| 153 | PIK3R2 | phosphoinositide-3-kinase regulatory subunit 2 [Source:HGNC Symbol;Acc:HGNC:8980] | 10956 | -0.425 | -0.2828 | Yes |
| 154 | ITGA8 | integrin subunit alpha 8 [Source:HGNC Symbol;Acc:HGNC:6144] | 11046 | -0.437 | -0.2828 | Yes |
| 155 | VEGFD | vascular endothelial growth factor D [Source:HGNC Symbol;Acc:HGNC:3708] | 11095 | -0.443 | -0.2795 | Yes |
| 156 | PIK3R1 | phosphoinositide-3-kinase regulatory subunit 1 [Source:HGNC Symbol;Acc:HGNC:8979] | 11099 | -0.443 | -0.2726 | Yes |
| 157 | LAMB1 | laminin subunit beta 1 [Source:HGNC Symbol;Acc:HGNC:6486] | 11135 | -0.448 | -0.2681 | Yes |
| 158 | GSK3B | glycogen synthase kinase 3 beta [Source:HGNC Symbol;Acc:HGNC:4617] | 11157 | -0.451 | -0.2625 | Yes |
| 159 | COL6A1 | collagen type VI alpha 1 chain [Source:HGNC Symbol;Acc:HGNC:2211] | 11204 | -0.457 | -0.2588 | Yes |
| 160 | PPP1R12A | protein phosphatase 1 regulatory subunit 12A [Source:HGNC Symbol;Acc:HGNC:7618] | 11259 | -0.465 | -0.2555 | Yes |
| 161 | VAV2 | vav guanine nucleotide exchange factor 2 [Source:HGNC Symbol;Acc:HGNC:12658] | 11413 | -0.489 | -0.2597 | Yes |
| 162 | KDR | kinase insert domain receptor [Source:HGNC Symbol;Acc:HGNC:6307] | 11454 | -0.496 | -0.2548 | Yes |
| 163 | THBS1 | thrombospondin 1 [Source:HGNC Symbol;Acc:HGNC:11785] | 11498 | -0.501 | -0.2502 | Yes |
| 164 | ITGA2B | integrin subunit alpha 2b [Source:HGNC Symbol;Acc:HGNC:6138] | 11645 | -0.524 | -0.2532 | Yes |
| 165 | PPP1CB | protein phosphatase 1 catalytic subunit beta [Source:HGNC Symbol;Acc:HGNC:9282] | 11737 | -0.539 | -0.2517 | Yes |
| 166 | PTEN | phosphatase and tensin homolog [Source:HGNC Symbol;Acc:HGNC:9588] | 11836 | -0.556 | -0.2505 | Yes |
| 167 | PDPK1 | 3-phosphoinositide dependent protein kinase 1 [Source:HGNC Symbol;Acc:HGNC:8816] | 11839 | -0.557 | -0.2417 | Yes |
| 168 | COL4A2 | collagen type IV alpha 2 chain [Source:HGNC Symbol;Acc:HGNC:2203] | 11940 | -0.580 | -0.2402 | Yes |
| 169 | SOS1 | SOS Ras/Rac guanine nucleotide exchange factor 1 [Source:HGNC Symbol;Acc:HGNC:11187] | 12041 | -0.603 | -0.2383 | Yes |
| 170 | ROCK1 | Rho associated coiled-coil containing protein kinase 1 [Source:HGNC Symbol;Acc:HGNC:10251] | 12046 | -0.605 | -0.2289 | Yes |
| 171 | PDGFA | platelet derived growth factor subunit A [Source:HGNC Symbol;Acc:HGNC:8799] | 12104 | -0.621 | -0.2234 | Yes |
| 172 | EGFR | epidermal growth factor receptor [Source:HGNC Symbol;Acc:HGNC:3236] | 12109 | -0.622 | -0.2137 | Yes |
| 173 | PDGFB | platelet derived growth factor subunit B [Source:HGNC Symbol;Acc:HGNC:8800] | 12389 | -0.704 | -0.2243 | Yes |
| 174 | FLT4 | fms related receptor tyrosine kinase 4 [Source:HGNC Symbol;Acc:HGNC:3767] | 12396 | -0.705 | -0.2134 | Yes |
| 175 | LAMA3 | laminin subunit alpha 3 [Source:HGNC Symbol;Acc:HGNC:6483] | 12449 | -0.724 | -0.2059 | Yes |
| 176 | ITGA5 | integrin subunit alpha 5 [Source:HGNC Symbol;Acc:HGNC:6141] | 12474 | -0.737 | -0.1959 | Yes |
| 177 | COL6A3 | collagen type VI alpha 3 chain [Source:HGNC Symbol;Acc:HGNC:2213] | 12506 | -0.750 | -0.1863 | Yes |
| 178 | ITGB1 | integrin subunit beta 1 [Source:HGNC Symbol;Acc:HGNC:6153] | 12566 | -0.780 | -0.1784 | Yes |
| 179 | ACTG1 | actin gamma 1 [Source:HGNC Symbol;Acc:HGNC:144] | 12613 | -0.806 | -0.1690 | Yes |
| 180 | VEGFA | vascular endothelial growth factor A [Source:HGNC Symbol;Acc:HGNC:12680] | 12629 | -0.813 | -0.1571 | Yes |
| 181 | CDC42 | cell division cycle 42 [Source:HGNC Symbol;Acc:HGNC:1736] | 12681 | -0.852 | -0.1474 | Yes |
| 182 | IGF1R | insulin like growth factor 1 receptor [Source:HGNC Symbol;Acc:HGNC:5465] | 12689 | -0.855 | -0.1342 | Yes |
| 183 | LAMA5 | laminin subunit alpha 5 [Source:HGNC Symbol;Acc:HGNC:6485] | 12692 | -0.859 | -0.1205 | Yes |
| 184 | COL4A1 | collagen type IV alpha 1 chain [Source:HGNC Symbol;Acc:HGNC:2202] | 12703 | -0.866 | -0.1074 | Yes |
| 185 | ACTB | actin beta [Source:HGNC Symbol;Acc:HGNC:132] | 12757 | -0.920 | -0.0967 | Yes |
| 186 | JUN | "Jun proto-oncogene, AP-1 transcription factor subunit [Source:HGNC Symbol;Acc:HGNC:6204]" | 12813 | -0.994 | -0.0850 | Yes |
| 187 | FN1 | fibronectin 1 [Source:HGNC Symbol;Acc:HGNC:3778] | 12820 | -1.005 | -0.0694 | Yes |
| 188 | PGF | placental growth factor [Source:HGNC Symbol;Acc:HGNC:8893] | 12850 | -1.059 | -0.0546 | Yes |
| 189 | RAPGEF1 | Rap guanine nucleotide exchange factor 1 [Source:HGNC Symbol;Acc:HGNC:4568] | 12860 | -1.098 | -0.0376 | Yes |
| 190 | CTNNB1 | catenin beta 1 [Source:HGNC Symbol;Acc:HGNC:2514] | 12866 | -1.119 | -0.0200 | Yes |
| 191 | AKT2 | AKT serine/threonine kinase 2 [Source:HGNC Symbol;Acc:HGNC:392] | 12896 | -1.404 | 0.0003 | Yes |
Table: GSEA details [plain text format]

  

Fig 2: KEGG\_FOCAL\_ADHESION      
 Blue-Pink O' Gram in the Space of the Analyzed GeneSet

  

Fig 3: KEGG\_FOCAL\_ADHESION: Random ES distribution      
 Gene set null distribution of ES for **KEGG\_FOCAL\_ADHESION**
